# Consequences of shade management on the taxonomic patterns and functional diversity of termites (Blattodea: Termitidae) in cocoa agroforestry systems

**DOI:** 10.1002/ece3.4607

**Published:** 2018-11-10

**Authors:** Ambele C. Felicitas, Bisseleua D. B. Hervé, Sunday Ekesi, Komivi S. Akutse, Christian T. C. L. Djuideu, Marie J. Meupia, Olubukola O. Babalola

**Affiliations:** ^1^ International Centre of Insect Physiology and Ecology (icipe) Nairobi Kenya; ^2^ Food Security and Safety, Faculty of Agriculture, Science and Technology North‐West University Mmabatho South Africa; ^3^ World Cocoa Foundation Accra Ghana; ^4^ University of Yaoundé 1 Yaoundé Cameroon

**Keywords:** agricultural intensification, ecosystem services, functional diversity, shade management, termites

## Abstract

Termites have gained importance as major pests in cocoa agroforests. Proper identification of termite species and knowledge on their functional diversity are the first steps in developing environmentally compatible management strategies. We tested the hypothesis that patterns of termite species richness in different cocoa agroforests is related to responses by termite functional groups to changes in shade management. We compared termite assemblages under five cocoa agroforestry systems in Cameroon to assess the impact of shade on termite taxonomic and functional group diversity. Sampling was done using a modified standardized transect method. Two 30 × 30 m quadrates each divided into three transects were laid in four farms at each site. Termites sampled were identified and grouped according to habitats, functional groups, and feeding habits. Sixty‐nine termite species in 33 genera and five subfamilies under two families were sampled. Termitidae was the most dominant family and Rhinotermitidae the least dominant with few species. Termite species richness decreased significantly from the heavy shaded cocoa agroforests (44 species) to the full sun (11 species). Functional group pattern differed significantly in all the cocoa agroforests and within each agroforestry system and dominated by wood and litter feeder species. Many species belonging to this group were responsible to most damages on cocoa trees. Both the richness of termite pests and marketable yield followed a quadratic curve and were found to be lowest and highest in plots with shade cover above 40%. The simulated optimal shade levels for low termite infestations and marketable yield overlapped between 45% and 65% indicating that cocoa agroforestry systems with around 55% shade cover may be optimal to balance termite infestations and marketable yield. Shade maintenance in cocoa agroforests is valuable in reducing termite pest species and conserving soil feeding termites which provide beneficial ecosystem services.

## 1 INTRODUCTION

Cocoa (*Theobroma cacao* L.) is shade‐tolerant and traditionally grown under shade trees in complex agroforestry systems, thereby providing a refuge for biodiversity and sustaining other ecosystem services (Bisseleua, Missoup, & Vidal, 2009; Rice & Greenberg, 2000). In spite of its ecological benefits, recent decades have seen a transformation of cocoa farming in southern Cameroon to more intensified systems by eliminating shade trees to increase short‐term income. This has resulted in a broad range of cocoa plantation management, ranging from low‐input shaded plantations to high‐input full sun plantations (Bisseleua, Fotio, Yede, & Vidal, 2013). It is assumed that shaded plantations are less profitable, but this assumption is often based on incomplete cost‐benefit calculations (Tondoh et al., 2015). This is because cocoa productivity is often used as an indicator for profitability, which is assumed to be lower for shaded systems. For example, there have been recent studies demonstrating the biodiversity benefits of shaded cocoa systems, with foci on ants, wasps, spiders, and plant diversity and how to manage farms to optimize diversity (Bisseleua & Vidal, 2011; Bisseleua et al., 2013). However, these studies documented a loss of biodiversity at the transition from shaded agroforestry systems to unshaded monocultures (Bisseleua et al., 2013, 2009 ) with the likeliness changing in ecosystem services resulting in higher incidences of pest outbreaks, such as termites (Bisseleua & Vidal, 2011). In an agroforestry trial in southern Cameroon, Dibog, Eggleton, Norgrove, Bignell, and Hauser (1999) demonstrated that tree canopy cover was positively correlated with termite abundance. This implies that agroforestry systems with more shade trees are habitat for more species, many of which actually help to increase the yield of cocoa, such as termites, ants, and other pollinators.

In cocoa agroforestry systems of West Africa, termites have long been associated with cocoa (Ambele, Bisseleua Daghela, Babalola, & Ekesi, 2018; Eggleton et al., 2002; Tra Bi, Soro, Yéboué, Tano, & Konaté, 2015). They are major soil‐dwelling ecosystem providers that influence ecosystem functions by changing their biotic and abiotic surroundings (Jouquet, Blanchart, & Capowiezc, 2014). They contain species that may become pests where cocoa is gradually grown in less‐shaded plantations or in full sun. However, they are far more beneficial through soil turnover during their foraging and nesting activities; facilitate soil aeration, enhancing absorption and storage of water including carbon fluxes and storage. An assessment of their damages in cocoa plantations of West Africa revealed that 20–80% of cocoa, specifically seedlings, were sometimes damaged to the extent of requiring replacement (Tra Bi et al., 2015). They are known to feed on bark, branches, and, sometimes, cocoa pods and to build many galleries on the stem from the base to the branches (Ambele et al., 2018; Tra Bi et al., 2015).

Although cocoa is important for national macroeconomic balances and provides livelihoods to millions of people in developing and developed countries, no studies have looked at the link between the biodiversity of termites and the functioning of cocoa agroforestry systems. Understanding termite functional diversity in cocoa agroforests is thus a very important initial step to developing realistic compatible management strategies against the pest species in cocoa agroforests. However, since species richness is also commonly used as the main measure of biodiversity, biological diversity concept that also includes taxonomic and functional diversity needs to be explored (Nunes et al., 2017; Pavoine & Bonsall, 2011). It is therefore paramount to understand both termite taxonomic and functional diversity in cocoa agroforestry systems that can also improve our knowledge on biodiversity patterns and how different land‐use intensification influences termite species diversity and their traits, since they capture different aspects of termite ecological roles. It is also very important to recognize termite functional groups in cocoa agroforests in order to develop specific and effective control measures to the target group(s) causing damage on cocoa. The aim of this study was therefore to compare the termite taxonomic and functional diversity of five different cocoa agroforestry systems in the central region of Cameroon. We hypothesized that the degree of shade cover and management in cocoa agroforests are the main determinants of termite diversity and functional group patterns and thus postulate that termite diversity and functional groups would vary according to the different shade systems.

## 2 MATERIALS AND METHODS

### 2.1 Study sites

**Figure 1:**
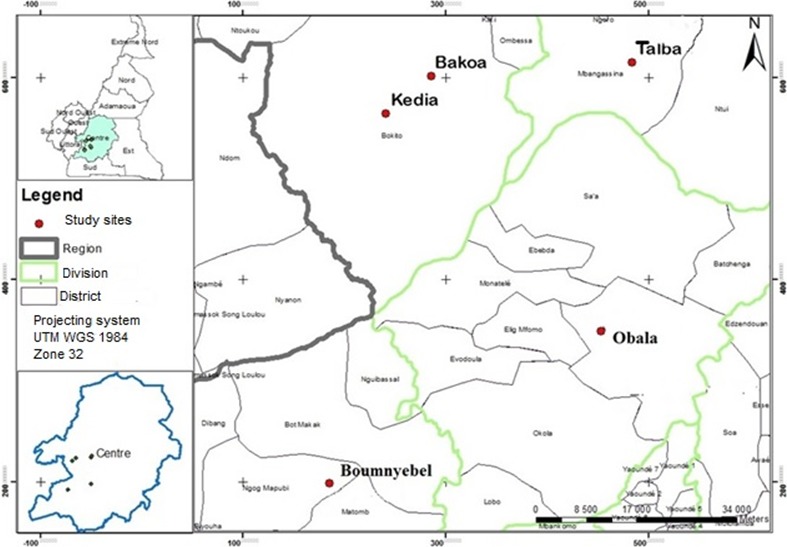
Map of the study areas in southern Cameroon

The study was conducted in five major cocoa‐growing areas in southern Cameroon located between 03°53′01″N and 4°56'42"N and 10°50′56″E and 11°16'47″E (Figure 1). The altitude varies between 402 m and 557 m above sea level (a.s.l.), characterized by a subequatorial climate with a dry season lasting over 3 months (December–March) during which the monthly rainfall is less than 70 mm and a rainy season lasting over 9 months (March–December), where the mean annual rainfall is about 1600 mm. The mean annual temperature is about 25°C with a relatively small thermal variation (Vidal, 2008). The pH of the soils varies from 4.29 to 5.43 (Kanmegne, Smaling, Brussaard, Gansop‐Kouomegne, & Boukong, 2006).

The five selected cocoa agroforestry (AF) systems are characterized as follows: (a) Boumnyebel (03°53′01″N 10°50′56″E, 402 m a.s.l.), where cocoa is grown under a dense cover of many forest tree species near pristine forests with very old cocoa plantation (30 years) on oxisols highly unsaturated acid rocks; (b) Obala (04°15′82″N 11°53′62″E, 557 m a.s.l.) located in semideciduous forest near houses, where cocoa is grown together with a high variety of fruit tree species, with no remnant forests because of very high human population density with relatively old cocoa plantations (20 years) on oxisols with fairly unsaturated acid rocks; (c) Talba (04°34'421"N 11°28'33"E, 462 m a.s.l.) where cocoa is grown in larger farms with mature cocoa plantations (30–35 years) in or near forests on ferric oxisols; (d) Kedia (4̊50'0.46"N, 11°07'87"E, 459 m a.s.l.) where young cocoa plantations (20–25 years) are grown under very low shade with young cocoa plantation in the savanna on ferric acrisols; (e) Bakoa (4°56'42"N 11°16'47"E, 469 m a.s.l.), where young cocoa plantation (20–25 years) is grown under full sun on modified savanna agroecosystems on ferric acrisols. The shade levels in the five AF systems were described by Bisseleua et al. (2013) with a little modification that more farms are gradually grown under full sun in Bakoa, and range from very low shade in Kedia, intermediate shade in Talba, moderate shade in Obala, and very heavy shade in Boumnyebel. The geographical coordinates of the selected AF systems were taken using a GPS (GPSMAP 60CSx). Termites were sampled in four cocoa plantations per system (20 farms in total). At each AF system, the selected plantations were distant for at least 500 m away from each other. Permissions for assessing the species richness and to conduct field studies within the cocoa plots were provided by the cocoa grower's associations from the selected regions. The field locations were privately owned by the farmers and did not harbor endangered or protected species. All cocoa farmers in the study area manage their farms in the same way despite differences in shade cover. Insecticides are directly sprayed on the cocoa pods, and weeding operations were applied once before flowering in all selected cocoa plantations.

### 2.2 Sampling methods

#### 2.2.1 Sampling of termites

We assessed termite species composition using a modified transect method to obtain a representative sample of both the taxonomic and functional group compositions of the local termite assemblage (Jones & Eggleton, 2000). We established two 30 × 30 m quadrates in each cocoa plantation, with a minimum distance of 50 m between the two quadrates. The quadrates were placed at least 30 m away from edges to avoid edge effect. We divided each quadrate into three transects of 30 × 2 m at the right, middle, and left of the quadrate. Each transect was further divided into 15 contiguous sections of 2 × 2 m each; making a total of 45 sections in each 30 × 30 m quadrate. In each section, we broke and opened all fallen dead woods of less than 1 m in diameter to remove termites. In addition, we also searched the leaf litter and dug and collected three soil samples of 12 cm ×12 cm and 10 cm deep with the aid of a machete and mixed to form one composite soil sample for each section. The soil was sieved and hand‐searched for termites with searching independent of time. Dead wood (diameter > 5 cm) within each of the quadrates (up to a height of 2 m) as well as galleries on cocoa trees were also searched for termites’ collection. Soldier and worker castes were collected and stored in 75% ethanol in 1.5‐ml Eppendorf tubes and labeled accordingly with the site, quadrate, and section numbers. The microhabitats (dead wood, litter, soil, and tree) where termites were encountered were also recorded simultaneously during the collections. Every species but not every termite specimen was collected for identification in the laboratory. Sampling was done only during the rainy season (June to October, 2016) because climatic seasons have been found not to significantly affect the termites’ fauna (Couto, Albuquerque, Vasconcellos, & Castro, 2015; Dibog, Eggleton, & Forzi, 1998).

#### 2.2.2 Sampling of vegetation and yield

In each selected plantation, we collected data on the vegetation characteristics. We recorded the number of shade trees species, with unknown tree species given a unique morphospecies number. We measured shade cover at 10 subpoints for each of the four cardinal directions within a 30 m radius circle using a convex spherical densitometer (R. E. Lemmon Forest Densiometers, USA) and calculated the mean shade cover per circle. Scientific and vernacular names (the latter given by local stakeholders) were recorded. Species that could not be identified in the field were identified at the National Herbarium of Cameroon (Yaoundé).

We assessed marketable yield in each quadrate by recording the number of flowers and young and mature cocoa pods (classified by size). Ripe pods were harvested, beans were pooled from the pods of one subplot, and dry weight of marketable beans was recorded. To account for yield, we separated defective beans and weighed them separately from marketable dry beans.

### 2.3 Termite identifications

Collections of soldier castes were identified to genus and sometimes to species levels using appropriate dichotomous keys (Bouillon & Mathot, 1965; Emerson, Lang, Chapin, & Bequaert, 1928; Sands, 1972, 1998 ), and through the reference collections of Institut de Recherche Agricole pour le Développement (IRAD) Nkolbisson, Yaoundé (Cameroon) with the technical support of Dr. Luc Dibog (Termite Taxonomist) of the Laboratory of Entomology of IRAD. Whenever possible specimens were identified to species level, and when this proved impossible, numbers (e.g., *Microtermes* sp. 1) were used to refer to unidentified species because of the complexity of Macrotermitinae taxonomy, and many species are not easy to be identified with certainty (Darlington, Benson, Cook, & Walker, 2008). New morphospecies were assigned numbers that followed the existing numbers in each genus. The identified specimens were placed in 75% ethanol in 1.5‐ml Eppendorf tubes with labels (name of species, locality, farm, name of identifier, date identified) inserted into each tube. The tubes with the specimens are deposited in the Zoological Collections of the Entomology Laboratory of IRAD, Nkolbisson, Yaoundé (Cameroon).

During identification, the enteric valve (a reduced proctodeal segment (P2 homolog) that is located between the first proctodeal segment (P1) and the third proctodeal segment (P3) of the digestive tube) was dissected and used to identify soldierless termites. Prior to their dissection, the specimens were immersed in 75% ethanol and dissected under a Huvitz dissecting microscope using entomological mandrins. The abdominal integument was removed from each specimen, and the mandrin was used to cut out the section of the gut containing part of the P1, P2 (containing the enteric valve), and P3. The gut section was placed on a microscopic slide in a drop of Berlese mounting liquid, and the organic matter from inside the gut section and muscle tissue from the outer wall of the gut were carefully removed. The gut section was then cut in half and splayed out on another microscope slide in another drop of Berlese mounting liquid. After re‐orientating the enteric pads and longitudinal ridges to face upwards on the slide, a drop of Berlese mounting liquid was added and a cover slip carefully placed on the slide. The slides were then observed under an Olympus microscope at a magnification of 100 xs. The enteric valve structures were compared to previous features using Sands (1972, 1998 ) or to existing prepared reference slides preparations of enteric valves structures of termite collections from Mbalmayo forest reserve in Cameroon stored at IRAD.

### 2.4 Functional and nesting groups

After identification, the termite species were assigned to feeding guilds or functional groups based on known feeding habits (Constantino, 1992; Donovan, Eggleton, & Bignell, 2001; Eggleton et al., 1996). These groups are as follows:

Group I: Wood and grass feeders (lower termites);
Group II: Wood and litter feeders (termites with a range of feeding habits) with many species responsible to feeding damages to cocoa (Ackonor, 1997; Tra Bi et al., 2015; Vos, Ritchie, & Flood, 2003);
Group III: Termites feeding on the organic rich upper layers of the soil (humus feeders);
Group IV: True soil feeders, ingesting apparently mineral soil.
Termites were also grouped into one of the five nesting groups (Bignell & Eggleton, 2000): hypogeal nesters that live below the ground; epigeal nesters which have colony centers above the ground (excluding arboreal nests); arboreal nesters that are found at different heights fixed to trees; log nesters that are normally found within dead logs or standing trees; and the hypo‐epigeal nesters which have their colony centers partly below and partly above the ground (Isra et al., 2008).

### 2.5 Analyses

The presence of a species in a section was used as a surrogate for relative abundance (Davies et al., 2003). In order to confirm sufficient sampling efforts for each site, the first‐order jackknife estimator was utilized, using EstimateS (Version 9.1.0) (Colwell, 2013), with 500 randomizations without replacing the samples. The first‐order jackknife estimator is considered to be the best estimator of nonparametric species richness (Basualdo, 2011). To compare species richness between localities, we constructed rarefaction curves by randomly simulating 500 curves based on the initial data from each transect. The overall spatial autocorrelation between environmental and response variables (species richness and occurrence) of the cocoa farms was determined by generating spatial correlograms. In so doing, we plotted similarity (Moran's I) of data points (i and j) as a function of the distance between cocoa farms (dij) to generate the Moran's I correlograms (Legendre & Legendre, 1998) using PASSaGE (Pattern Analysis, Spatial Statistics and Geographic Exegesis, version 2; Rosenberg & Anderson, 2011). The analysis also included a Mantel test with 999 permutations to establish significance (α = 0.05) for the pairwise determination of spatial autocorrelation between the study sites (Legendre & Legendre, 1998). We used generalized least squares (GLS) model to correct for spatial autocorrelation and analyze for the differences between species richness in the selected cocoa agroforestry types (Dormann et al., 2007). This analysis was performed using the R software, version 3.2.3 (R Development Core Team, 2015). We also categorized the termite species sampled into two groups: the pest and non‐pest species, and we analyzed the differences observed between AF systems using one‐way ANOVA. The percent shade cover between sites was also analyzed with ANOVA. Prior to performing ANOVA, the assumption of homogeneity of variance was tested and satisfied using Bartlett's test. In case of significance differences, means were separated with Student–Newman–Keuls (SNK) post hoc test. In data cases where the assumptions of ANOVA were violated (variances not homogenous), the nonparametric Kruskal–Wallis tests was conducted with a post hoc Dunn test performed, where *p*‐values were adjusted using the Benjamini–Hochberg method. Replicate transects were pooled for these analyses. The Shannon–Wiener diversity index (H́) was further used to compare the species richness and evenness between the five cocoa agroforestry types.

To describe beta‐diversity or spatial turnover, a pairwise comparison between the five shade types was computed using the Jaccard's index (JI) of similarity. We compared the functional group occurrence between the different localities using nonparametric Kruskal–Wallis tests with a post hoc Dunn test pairwise comparison in R software. This is because functional group occurrence data were not normally distributed and could not be normalized by transformation. The functional evenness was also processed with the conventional species diversity index (Shannon–Wiener index), since the information on species’ assignment to functional groups was available and easy to obtain and needed a low level of detail in contrasting species traits (Schleuter, Daufresne, Massol, & Argillier, 2010; Stevens, Cox, Strauss, & Willig, 2003).

Based on the feeding group classification, a weighted humification score (HS) was used to compute the community weighted mean (CWM) for each functional group. The HS depicts the position of termite species along a gradient of increasing humification of their food substrate (Davies et al., 2003; Donovan et al., 2001), and the weighted HS depicts the position of the functional groups along this gradient. The CWM was therefore calculated following Garnier et al. (2004):

$$\text{CWM}=\sum_{i=1}^{N}p_{i}f_{i}$$

where *p_i_* is the relative abundance of functional group *i*,* f_i_* is the corresponding termite feeding group score, ranging from *f* = 1 for wood and grass feeders (Group I) to *f* = 4 for true soil feeders (Group IV), and *N* is the total number of termite encounters per functional group per locality.

## 3 RESULTS

### 3.1 Taxonomic diversity

We recorded 69 termite species belonging to two families; Termitidae and Rhinotermitidae (subfamily: Coptotermitinae). In all the localities, the family Termitidae comprising four subfamilies (Table 2) was dominant in terms of richness and number of times recorded. Rhinotermitidae was represented by only a single species (*Coptotermes sjoestedti*) recorded at one farm in one locality (Kedia). Analysis of Moran's I indicated significant autocorrelation for both species richness and species abundance. The pairwise comparison of sites with Mantel test indicated spatial autocorrelation between Boumnyebel and Obala (*r* = −0.57, *p* = 0.22), Boumnyebel and Kedia (*r* = 0.11, *p* = 0.8), Boumnyebel and Bakoa (*r* = −0.37, *p* = 0.43), Obala and Talba (*r* = −0.44, *p* = 0.34), Obala and Kedia (*r* = 0.02, *p* = 0.95), Obala and Bakoa (*r* = −0.49, *p* = 0.36), Talba and Kedia (*r* = 0.09, *p* = 0.82), Talba and Bakoa (*r* = −0.49, *p* = 0.29), and Kedia and Bakoa (*r* = −0.28, *p* = 0.34), but no evidence of spatial autocorrelation was shown between Boumnyebel and Talba (*r* = 0.94, *p* = 0.04).

**Figure 2:**
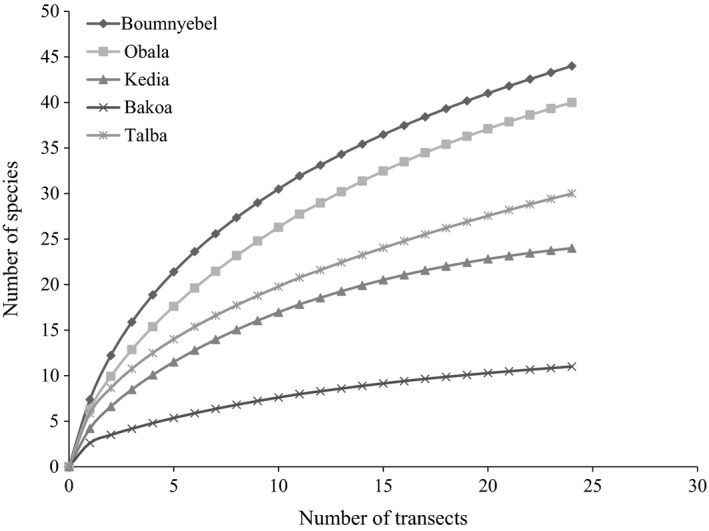
Rarefaction curves of termites sampled in five cocoa agroforestry systems in southern Cameroon

**Figure 3:**
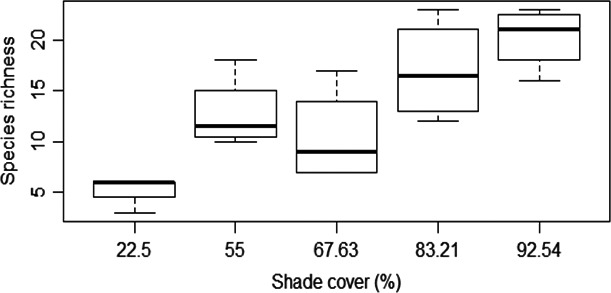
Community beta‐diversity of termite in relation to shade management in cocoa agroforestry systems of southern Cameroon

The rarefaction curves (Figure 2), the Shannon index (Table 1), and the GLS model results showed significant differences (*F* = 9.3, *p* = 0.0001, *df* = 4) between the species richness of termites in the five AF systems, with more species recorded in Boumnyebel (very heavy shade), followed by Obala (moderate shade), Talba (intermediate shade), Kedia (light shade), and the least in Bakoa (full sun) (Figure 3). Pairwise comparison of the different sites showed no significant differences in species richness of termites between Boumnyebel (20.25 ± 1.89; mean ± SE, *F* = 9.39, *df* = 4) and Obala (17 ± 1.89), Talba (12.75 ± 1.89) and Kedia (10.50 ± 1.89), or Kedia (10.50 ± 1.89) and Bakoa (5.25 ± 1.89) but significant differences in species richness were, however, observed between Boumnyebel (20.25 ± 1.89) and Talba (12.75 ± 1.89), Boumnyebel (20.25 ± 1.89) and Kedia (10.5 ± 1.89), Boumnyebel (20.25 ± 1.89) and Bakoa (5.25 ± 1.89), and Talba (12.75 ± 1.89) and Bakoa (5.25 ± 1.89).

**Table 1:** List of the 69 termite species collected from the five cocoa agroforestry systems in Cameroon and classified according to their functional, nesting, and feeding groups. Feeding groups: W: wood feed; W/L/P: wood and litter feeding including feeding on cocoa live plant; W/S: wood/soil feeding; (F): fungus growing; S: soil feeders

Species	Functional group	Nesting group	Feeding group	Species occurrence at the various sites				
				Boumnyebel	Obala	Kedia	Bakoa	Talba
Rhinotermitidae: Coptotermitinae								
*Coptotermes sjostedti* Holmgren	II	Hypogeal	W/P			2		
Termitidae: Macrotermitinae								
*Microtermes* sp. 1	II	Hypogeal	W/L/P (F)	82	256	144	184	341
*Microtermes* sp. 2	II	Hypogeal	W/L/P (F)	52	106	12	3	35
*Microtermes* sp. 3	II	Hypogeal	W/L/P (F)	3	2			
*Ancistrotermes crucifer* (Sjoestedt)	II	Hypogeal	W/L/P	81	18	111	161	134
*Ancistrotermes* sp. 1	II	Hypogeal	W/L/P		1			
*Macrotermes bellicosus* (Smeathman)	II	Hypo‐epigeal	W/L/P		12	1		2
*Macrotermes lilljeborgi* (Sjoestedt)	II	Hypogeal	W/L/P		1	1	2	2
*Odontotermes culturarum* Sjoestedt	II	Hypogeal	W/L/P			6		6
*Odontotermes silvaticus* Harris	II	Hypogeal	W/L/P					1
*Odontotermes bequaerti* Emerson	II	Hypogeal	W/L/P			2		3
*Odontotermes schmitzi*	II	Hypogeal	W/L/P	9		9		3
*Pseudacanthotermes militaris* (Hagen)	II	Hypo‐epigeal	W/L/P (F)		27	31	6	
*Sphaerotermes sphaerothorax* Holmgren	II	Hypogeal	W	5	2			9
*Protermes prorepens*	II	Hypogeal	W		2			
*Synacanthotermes heterodon*	II	Hypogeal						2
Termitidae: Termitinae								
Termes group								
*Pericapritermes nigerianus*	III	Hypogeal	S	5		3		
*Pericapritermes* sp. nov. 1	III	Hypogeal	S	1	2			
*Promirotermes orthoceps*	III	Hypogeal	S				1	2
*Profastigitermes putnami* Emerson	IV	Hypogeal	S	5	3	3	2	
*Fastigitermes jucundus* (Sjoestedt)	IV	Hypogeal	S	3	3	5		5
*Probosciterme*s *tubuliferus*	IV	Hypogeal	S	3	1	1		2
Cubitermes group								
*Apilitermes longiceps* Holmgren	IV	Hypogeal	S	1				
*Apilitermes* sp. nov. 1	IV	Hypogeal	S	2				
*Basidentitermes malelaensis* (Emerson)	IV	Hypogeal	S	4				
*Cubitermes severus*	IV	Hypogeal	S	7				
*Furculitermes winifredi* Emerson	IV	Hypogeal	S	1				
Amitermes group								
*Microcerotermes fuscotibialis* (Sjoestedt)	II	Arboreal	W	26	6			19
*Microcerotermes parvus* (Haviland)	II	Arboreal	W	32	30		1	1
*Microcerotermes edentatus* Wasmann	II	Hypogeal	W	18	3			2
*Microcerotermes progrediens*	II	Hypo‐epigeal	W	13	8			23
*Microcerotermes sylvestrianus*	II	Hypo‐epigeal	W	4				
Termitidae: Nasutitermitinae								
*Nasutitermes arborum* (Smeathman)	II	Arboreal	W/P			7		8
*Nasutitermes eleganthulus* (Sjoestedt)	II	Arboreal	W/P	1		7		
*Nasutitermes diabolus* ((Sjöstedt)	II	Arboreal	W/P			5	1	
*Trinervitermes carbonarius* Sjöstedt	I	Arboreal	G	1		3		
*Trinervitermes roseni* (Holmgren)	I	Arboreal		2	2			1
Termitidae: Apicotermitinae								
Anoplotermes group								
*Adaiphrotermes choanensis (Fuller)*	III	Hypogeal	S	12	1			
*Adaiphrotermes cuniculator Sands*	III	Hypogeal	S	1				
*Aderitotermes fossor* Sands	III	Hypogeal	S	3			2	2
*Aderitotermes cavator* Sands	III	Hypogeal	S					1
*Aderitotermes* sp. nov. 1	III	Hypogeal	S	2	4			
*Alyscotermes trestus* Sands	III	Hypogeal	S		1			
*Alyscotermes kilimandjaricus* (Sjoestedt)	III	Hypogeal	S		2	2		1
*Alyscotermes* sp. nov. 1	III	Hypogeal	S	1				
*Amalotermes phaeocephalus* Sands	III	Hypogeal	W/S	1	2			6
*Amalotermes* sp nov. 4	III	Hypogeal	S					1
*Amicotermes galenus* Sands	IV	Hypogeal	S		1			1
*Amicotermes* sp. nov. 1	IV	Hypogeal	S		3			
*Amicotermes* sp. nov. 2	IV	Hypogeal	S	2	1			
*Amicotermes* sp. nov. 3	IV	Hypogeal	S		1			2
*Amicotermes* sp. nov. 4	IV	Hypogeal	S		1			4
*Anenteotermes polyscolus* Sands	IV	Hypogeal	S	9	16	1	4	10
*Anenteotermes* sp. nov. 1	IV	Hypogeal	S	2				
*Anenteotermes cnaphorus*	IV	Hypogeal	S	1				
*Anenteotermes ateuchestes* Sands	IV	Hypogeal		1				
*Astalotermes amicus* Sands	III	Hypogeal	S	4	2			1
*Astalotermes quietus* (Silvestri)	III	Hypo‐epigeal	S	8	6			
*Astalotermes empodius* Sands	III	Hypogeal	S	1	1			
*Astalotermes* sp. nov. 1	III	Hypogeal	S	1	4			
*Astalotermes* sp. nov. 2	III	Hypogeal	S	1				
*Astratotermes aneristus* Sands	III	Hypogeal	S		1			
*Astratotermes pacatus* (Silvestri)	III	Hypogeal	S	1	1			
*Astratotermes prosenus* Sands	III	Hypogeal	S	2	1			
*Ateuchotermes ctenopher* (Sands)	IV	Hypogeal	S	1	5			
Apicotermes group								
*Duplidentitermes furcatidens* (Sjoestedt)	IV	Hypogeal	S		2	2		
*Eburinitermes* sp.	IV	Hypogeal	S	3				
Apicotermitinae new genus	IV	Hypogeal	S			2		
Total number of encounters				416	542	365	367	628
Observed number of species (*S*_obs_)				44	40	24	11	30
Expected richness (jackknife 1 (mean ± SE))				60.29 ± 0.46	55.33 ± 0.52	29.75 ± 0.30	14.83 ± 0.22	43.42 ± 0.45
Sampling effort				73%	72%	81%	74%	70%
Shannon–Wiener index (*H*')				2.76	2.03	1.87	1.00	1.65
Functional evenness (*H*')				0.71	0.48	0.30	0.11	0.27

We noted that from the 44 termite species recorded in Boumnyebel, eleven (11) species (16.18%) were endemic to this locality. In Obala, we recorded 40 species of which five (7.35%) were endemic, while in Talba, four (5.88%) species out of the 30 species recorded were endemic to this AF system. In Kedia, we recorded 24 species with only two (2.94%) endemic species. All the 11 termite species recorded in Bakoa were also found in other AF systems (Table 1). We observed that five termite species (*Ancistrotermes crucifer*,* Anenteotermes polyscolus*,* Microcerotermes parvus*,* Microtermes* sp. 1 and *Microtermes* sp. 2) from the 69 identified were recorded across all the five AF systems with *Microtermes* sp. 1 scoring the highest number of specimens (211.4 ± 50.05), followed by *Ancistrotermes crucifer* (101 ± 50.05) and *Microtermes* sp. 2 (41.2 ± 50.05) (Table 1).

**Figure 4:**
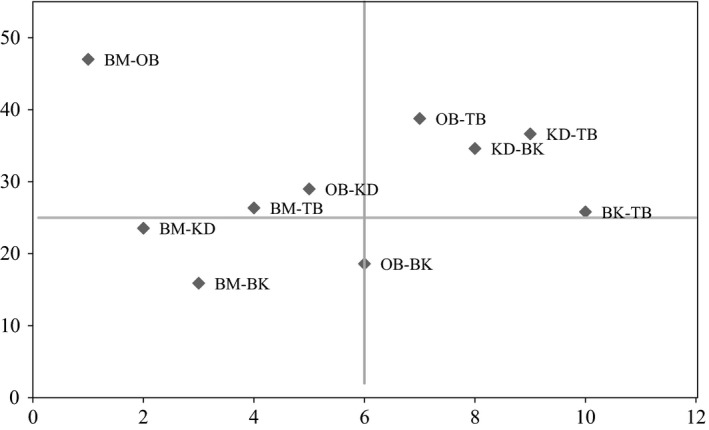
Jaccard similarity index (%) of cocoa agroforestry systems (BM: Boumnyebel; OB: Obala; TB: Talba; KD: Kedia; BK: Bakoa)

We recorded an overall Jaccard index (JI) between the five AF systems of 7.35%. We found the highest species similarity between Boumnyebel and Obala (47%) and the lowest between Boumnyebel and Bakoa (15.91%) (Figure 4). The genera *Adaiphrotermes, Astratotermes,* and *Ateuchotermes* (all soil termites) were only found in Boumnyebel and Obala, whereas *Nasutitermes diabolus* was only found in Kedia and Bakoa.

### 3.2 Functional group diversity

The species composition per functional group in the different AF systems varied significantly (*H* = 57.28, *p* ˂ 0.001). Group II termites recorded the highest number of termites (90%), followed by Group IV (5%), Group III (4%), and Group I (1%). Group I termites were represented by only one genus (*Trinevitermes*), while Group II were represented by 11 genera with four genera (*Ancistrotermes, Microtermes, Microcerotermes,* and *Macrotermes*) present in all the AF systems (Table 2). Termites from Group II were the most frequently found functional group in Boumnyebel recorded 325 times (77.57%) out of the 419 total times recorded. This group in Boumnyebel was dominated by the genera *Microtermes* collected at 137 points, followed by *Microceroterme*s (93 points) and then *Ancistrotermes* (82 points). Termites from Group IV were the second most abundant recorded in 47 points of excavation, and *Anenteotermes,* the most dominant genus recorded 13 times. The remaining genera were sparsely represented (Table 1). Termites in Group III from Boumnyebel were mainly dominated by the genera *Adaiphrotermes* and *Astallotermes*. Group I was represented only by one genus (*Trinervitermes*). Termites from Group II were dominant in Obala recorded 474 times out of the total 542 times recorded. *Microtermes* was also identified as dominant, with 364 times out of the 474 times recorded, followed by *Microcerotermes* recorded 47 times. Group III and IV were recorded 33 times each with the dominant species belonging to the genera *Amicotermes* and *Anenteotermes,* respectively (Table 1). Functional group I (*Trinervitermes*) was recorded only twice in Obala. Group II also dominated termites found in Talba with 591 (94.11%) out of the 628 times recorded. Again, *Microtermes* collected in 426 points was the dominant genus followed by *Ancistrotermes* (134) and *Microcerotermes* (40). Groups III and IV were dominated by *Amalotermes* and *Anenteotermes,* respectively, while *Trinervitermes* was collected only at one point. Group II also dominated the functional group diversity patterns in Kedia and Bakoa. In these AF systems, groups III and IV were less represented, while Group I was not recorded in Bakoa but represented only by a single genus *Trinervitermes* in Kedia. Like for species evenness, the functional groups in Boumnyebel were more even, followed by Obala, Kedia, Talba, and then Bakoa (Table 1).

**Table 2:** Termite pest and non‐pest species richness and occurrence (±SE of the mean) sampled in five cocoa agroforestry systems in southern Cameroon in 2016

Cocoa AF system	Shade cover (%)	Pest termite species richness	Pest termite species occurrence	Non‐pest termite species richness	Non‐pest termite species occurrence
Boumnyebel	92.54 ± 2.43 a	8.5 ± 1.7 a	82.25 ± 60.28 a	12.0 ± 1.82 a	23.00 ± 10.39 a
Obala	83.21 ± 1.42 b	8.75 ± 0.5 a	119 ± 60.61 a	8.50 ± 4.43 a	16.50 ± 14.06 ab
Talba	67.63 ± 5.28 c	9.5 ± 1.7 a	148 ± 49.91 a	3.75 ± 2.63 b	9.00 ± 7.62 ab
Kedia	55 ± 5.71 d	7.75 ± 2.8 a	86 ± 22.30 a	2.75 ± 3.09 b	5.25 ± 5.5 ab
Bakoa	22.5 ± 2.08 e	3.75 ± 1.2 b	90 ± 50.97 a	1.25 ± 0.50 b	1.75 ± 0.95 b

Note

Values within a column followed by the same letter are not significantly different (*p* ˂ 0.05, SNK test).

**Figure 5:**
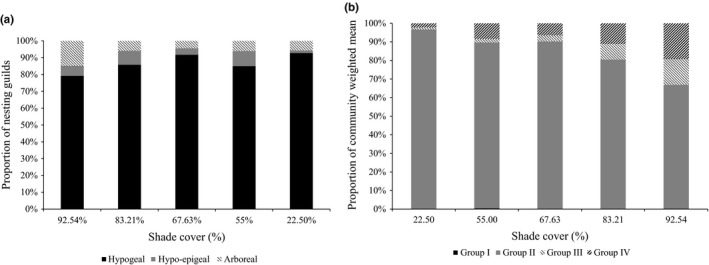
Nesting guilds (a) community weighted means (b) of the four functional groups of termite species sampled from cocoa agroforestry systems of southern Cameroon

The number of termites collected in the different microhabitats per AF system varied significantly (*F* = 6.7, *df* = 2, *p* = 0.01). Post hoc comparison with the SNK test showed that the mean number score of termites collected from soil (235.6 ± 38.48) was significantly higher than termites obtained in wood (132 ± 38.48) and litter (mean: 101.6 6 ± 38.48). From the 69 identified species, only 26 species were wood and litter feeders (groups I and II), while 43 species were soil feeders (groups III and VI). For the nesting guilds, most of the termite species sampled in the different sites were hypogeal, followed by hypo‐epigeal, while few species were arboreal termites (Figure 5a).

### 3.3 Effect of shade management on termites and yield

Of the total of 69 termite species sampled, 26 were pest species, while 43 were non‐pest species. Significant differences (*F* = 6.4, df = 4, *p* = 0.003) were observed in the species richness of pest species sampled from the five different AF systems. However, no significant differences (*F* = 1.22, df = 4, *p* = 0.34) were observed in the number of times (occurrence) the pest species were recorded from the five AF systems (Table 2). For the non‐pest species, significant differences (*F* = 10.04, df = 4, *p* = 0.0004) were observed in the species richness and also in the occurrence (*F* = 3.76, df = 4, *p* = 0.02) in the five AF systems (Table 2).

Richness of termite pest species was significantly lower under shade cover between 20% and 40%. The quadratic model showed a significant increase in the richness of pest species above 40% shade cover (*F* = 18.56, *R* ^2^ = 0.68; *p* < 0.0001). We noted a significant and positive relationship between shade cover and richness of beneficial termite species (e.g., non‐pest species) at shade cover >60% (*F* = 19.60, *R* ^2^ = 0.69; *p* < 0.0001) (see Figures S1–S3 and Table S1).

The community weighted means (CWM) results showed that functional group II (pest species) dominated in all the AF systems but with a decrease with increase in shade cover, while the functional groups III and IV (soil termites) increase with increase in shade cover (Figure 5b). The functional groups I and II (pest species) therefore had weaker correlation (*r* = 0.76) with percent shade cover than groups III and IV (soil termites) (*r* = 0.84). However, there was a weaker correlation (*r* = 0.2) of pest species occurrence and percent shade cover as compared to non‐pest species occurrence and percent shade cover (*r* = 0.71).

**Figure 6:**
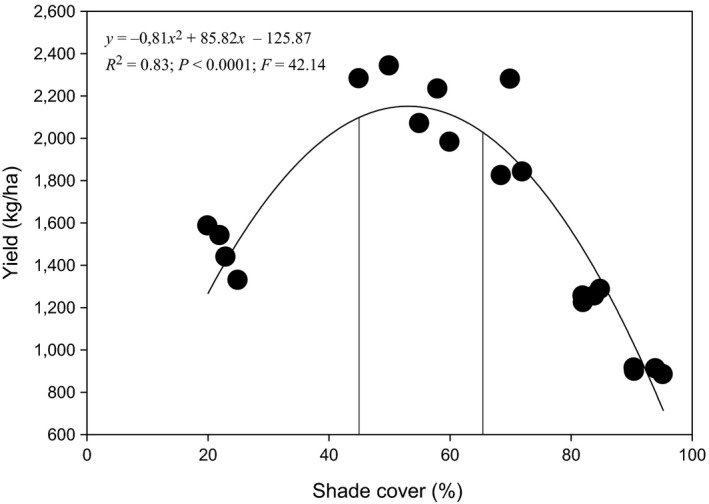
Relationship between yield and shade cover. Each point represents the mean value of all observation of 30 individual cocoa trees per plot over a 2‐year period. Vertical lines indicate optimal shade levels (solid)

Cocoa yield per AF system was significantly higher at optimal shade level between 45% and 65% after which yield decreased significantly (*F* = 42.14, *R* ^2^ = 0.83; *p* < 0.0001). The optimal shade levels to maintain a balance between the richness of termite pests and that of beneficial termites with cocoa yield overlapped between 45% and 65% (Figure 6).

## 4 DISCUSSION

We showed that shade management in cocoa agroforestry systems strongly impact termite species richness and composition because even though most of the AF systems were spatially autocorrelated, significant differences were still observed in the species diversity based on the differences in percent shade cover. Our data suggest that cocoa agroforestry systems with shade levels between 45% and 65% may be optimal to balance between richness of termite pest species and marketable yield and may help to increase the role of beneficial termites. These results are in line with shade levels recommended for good agronomic practices in cocoa cultivation (Asare & Raebild, 2016; Bisseleua et al., 2009).

The rustic cocoa agroforestry systems with heavy shade (92.54% shade cover) such as practiced in Boumnyebel revealed higher termite species richness and functional group diversity than the low shaded (67.63%) or full sun (22.2%) cocoa agroforestry systems. Soil‐feeding termites are strongly affected by shade removal than wood and litter feeders which are adapted to drier conditions. This was also seen with the CWM where many functional groups III and IV species were recorded in Bomneybel and Obala than the Kedia and Bokoa full sun cocoa AF systems. This suggests that when shade cover drops below a threshold, the soil‐feeder assemblages diminish.

Analysis from the species accumulation curves corroborated with the results from diversity indexes, with Boumnyebel having more termite species than Obala, Talba, Kedia, and Bakoa. However, Boumnyebel, Obala, and Talba curves showed no signs of reaching a plateau, although the rate of increase for Boumnyebel was higher than that of Obala, Talba, and Bakoa and the sampling effort was more than 70% for both AF systems. Previous ecological studies on the species richness of termites using same approach obtained similar curves, showing the difficulty to stabilize accumulation curves when sampling termites in places with high diversity (Carrijo, Brandão, Oliveira, Costa, & Santos, 2009; Constantino, 1992). According to Chao, Colwell, Lin, and Gotelli (2009), species accumulation curves have a disadvantage when large numbers of individuals must be sampled to reach the asymptote as high richness and rarity of many species makes estimation of sampling effort with accumulation curves difficult to predict. The sequence in species richness in the selected AF systems was (in decreasing order) Boumnyebel (heavy shaded system with 92.54% shade cover), Obala (moderate shade, 83.21%), Talba (intermediate shade, 67.63%), Kedia (light shade, 55%), and Bakoa (full sun, 22.2%). Previous studies on the effect of habitat fragmentation on the diversity of termite across forest landscape concluded that termite diversity decreases along the sequence from primary forest to secondary forest and food crop fields (Davies, 2002; de Souza & Brown, 1994). Other studies have shown that termite richness is higher in mature forest, intermediate in reforestation areas, and lower in secondary forest and pastures (de Paula, Silveira, Rocha, & Izzo, 2016). Since the rustic shade system of Boumnyebel and the moderate shade of Obala could be compared to secondary forest, and Kedia and Bakoa systems of very light shade and full sun cocoa to food crop fields, we can clearly see that the pattern of termite species richness observed in this study corroborates with previously reported results. Our results showed that removal of shade trees in cocoa plantations usually lead to hotter and drier microclimates that are not suitable for the survival of many soil‐feeding termites which are subterranean and soft bodied and therefore more sensitive to variation in microclimate (de Souza & Brown, 1994; Davies, Eggleton, Rensburg, & Parr, 2012). This implies that differences in shade management in cocoa agroforestry systems strongly impact termite species richness and composition.

The 11 termite species that were exclusively sampled in Boumnyebel and the four species that were exclusively found in Obala were all soil‐feeding termites. *Anenteotermes polyscolus* was the only soil termite recorded in all the AF systems. Apart from *A*. *polyscolus*, three other soil termite species *Promirotermes orthoceps* (recorded in Cameroon for the first time), *Profastigitermes putnami,* and *Aderitotermes fossor* were encountered in Bakoa under full sun cocoa production. Besides being the lowest locality in species richness, no species was endemic to Bakoa. In addition, no significant difference was observed in the pest species occurrence between the five AF systems. This implies that apart from the wood and litter feeders (Jones et al., 2003), many species are lost as shade cover decreases, but are not displaced by others. Contrary to our findings, Norgrove, Csuzdi, Forzi, Canet, and Gounes (2009) in their study of termite fauna in shaded cocoa plantations of southern Cameroon did not record *Apilitermes*,* Cubitermes, Microcerotermes,* and *Nasutitermes*. This could be due to the fact that the sampling methods differ in their emphasis on particular substrates, where in this study, the transect method used thoroughly sampled soil as well as leaf litter.

Termite species from the Termitidae family represented more than 95% of the total species recorded. Similar conclusion was made by Couto et al. (2015). These termite species easily adapt to disturbance because they are able to utilize other sources of cellulose in addition to wood (Inward, Vogler, & Eggleton, 2007) and hence their distribution in all the AF systems. The most common species of Termitidae encountered in all the AF systems included *Microtermes* spp., *Ancistrotermes* spp., and *Microcerotermes* spp. *Microtermes* and *Microcerotermes* were sampled on the truck and galleries on cocoa trees in Boumnyebel, Obala, and Talba cocoa agroforests, as well as on broken branches of cocoa hanging from the main branches, while *Ancistrotermes* were sampled on roots and soil around cocoa trees in all the AF systems. These species are known to be responsible for major damages on cocoa and companion trees (Mitchell, 2002; Pomeroy, Bagine, & Darlington, 1991). *Ancistrotermes* spp. feed on the root systems of young and mature cocoa trees, while *Microtermes* and *Microcerotermes* by building their galleries on cocoa trunk contribute to reduce flowers and pod production negatively affecting yield and productivity (Ackonor, 1997; Vos et al., 2003). Damages from *Ancistrotermes* and *Microtermes* species were more serious in cocoa farms at the forest–savanna transition zone (e.g., Kedia and Bakoa). These species have been reported to tolerate semiarid and even arid environmental conditions such as the one of the full sun and light shade systems where they are responsible for up to 80% seedling mortality following their transplantation (Rouland‐Lefïèvre & Mora, 2002). Therefore, their high abundance in cocoa agroforests should be a great concern to cocoa stakeholders, the cocoa industry, and the cocoa‐producing countries in particular.

Functional group composition was strongly correlated with variation in shade level, with members of functional groups III and IV (soil feeders) being most abundant in the shaded systems and rare in the low shade and full sun systems. This result is in line with previous findings from southern Cameroon, where tree canopy cover was positively correlated with termite abundance and species richness, with a decline in soil faunal population when trees were removed from land use systems (Dibog et al., 1999). Soil‐feeding termites have been shown to be vulnerable to habitat clearance, decreasing in species richness over wood feeders (Bandeira, Vasconcellos, Silva, & Constantino, 2003; Eggleton et al., 2002). Soil feeders are strongly affected by low shade, as they need stable conditions to fill their energetic limitations gaps (Isra et al., 2008). Functional groups I and II had weaker correlations with reduction in shade than functional groups III and IV. This observation could be attributed to the fact that they possess higher exoskeleton sclerotization which provides resistance to desiccation in open habitats such as Bakoa (full sun) and Kedia (light shade) (Luke, Fayle, Eggleton, Turner, & Davies, 2014). Other researchers have also found that wood feeders are more resilient to habitat conversion than soil feeders (Eggleton et al., 1996; 2002; Jones et al., 2003). Therefore, the significant decreases in occurrence of the soil feeders in Bakoa and Kedia could also be due to the fact that these termite groups are prone to desiccation (Gathorne‐Hardy, Syaukani Davies, Eggleton, & Jones, 2002). In Kedia and Bakoa, leaf litter and crop residues may also provide a year‐round available and abundant food resource for wood and litter feeders (Davies, 2002). This explains the abundance of *Ancistrotermes* and *Microtermes* species accounting for the dramatic increase in wood feeding termites in Bakoa and Kedia as compared to Bounmyebel, Obala, and Talba.

## 5 CONCLUSIONS

The results of our study conclusively showed that richness and evenness of termite species are driven by cocoa shade systems, with emphasis that (a) the number of termite species in the shaded systems was more diversified than that of the unshaded systems, demonstrating that shade management characteristics dictates termite faunal differences; (b) the shaded systems maintained all the termite species found in the full sun systems and harbored a diversity of non‐pest species, suggesting that the establishment of shade in cocoa agroforestry systems conserves important part of functional biodiversity of termites. In addition, (c) the majority of damages in cocoa agroforests is associated to members of the subfamilies Macrotermitinae and Termitinae. The high number of occurrence of members of these subfamilies across all the localities call for a need to develop adequate management strategies against these species and also to implement appropriate agricultural practices and extension programs targeting shade management and emerging pests such as termites in cocoa agroforestry systems. Furthermore, (d) soil‐feeding termites which create and maintain favorable soil conditions for plant growth and nutrient intake are more vulnerable to reduced shade cover over wood feeders which are becoming main pest of cocoa trees. (e) Shade reduction is also affecting wood feeders which are gradually shifting their food sources from leaf litter and crop residues to becoming main pest of cocoa trees feeding mainly on the root system. Our results indicate that cocoa agroforestry systems with around 55% to be optimal to balance termite infestations and marketable yield and should therefore be encouraged in cocoa production systems in order to create favorable conditions for the survival of soil‐feeding termites. Shaded cocoa agroforestry systems will maintain beneficial ecosystem services and minimize potential ecosystem disservices through trophic and non‐trophic interactions.

## CONFLICT OF INTEREST

None declared.

## AUTHOR CONTRIBUTIONS

BDHB and SE conceived and designed research. AFC, DTCL, and MMJ conducted the research. AFC analyzed the data. AFC, BDHB, SE, KSA, and BOB led the writing of the manuscript. All authors read the manuscript and gave final approval for publication.

## DATA ACCESSIBILITY

All authors agree to deposit all data supporting the results in this paper in an appropriate public repository such as Dryad.
