# Supplementary material for: Consequences of shade management on the taxonomic patterns and functional diversity of termites (Blattodea: Termitidae) in cocoa agroforestry systems
Source: Ecol Evol. 2018 Nov 10;8(23):11582–95. doi: 10.1002/ece3.4607 (PMC6303804; doi:10.1002/ece3.4607)
Supplement: Supplementary file 1 [file ECE3-8-11582-s001.docx]

**Supplementary documents**

**Table S1** Structural and functional characteristics of cocoa agroforestry systems in Southern Cameroon

|  | **Boumnyebel** | **Obala** | **Talba** | **Kedia** | **Bakoa** |
| --- | --- | --- | --- | --- | --- |
| Number of tree species (n) | 77 | 19 | 18 | 13 | 13 |
| Tree species richness | 10.81 ± 1.0a | 5.81 ± 0.61b | 3.5 ± 0.49c | 4.0 ± 1.24bcd | 5.0 ± 0.46bd |
| Tree density (ha) | 196.5 ± 15.5a | 158.3 ± 21.6ac | 65.9 ± 9.15b | 98.6 ± 31.8bc | 140.3 ± 31.4c |
| Total tree height (m) | 12.67 ± 0.47a | 12.92 ± 0.62a | 17.91 ± 0.96b | 8.6 ± 0.73c | 12.58 ± 0.63a |
| Shade cover (%) | 92.54 ± 2.43a | 83.21 ± 1.42b | 67.63 ± 5.28c | 55.00 ± 5.71d | 22.5 ± 2.08e |

Values within a row followed by the same letter are not significantly different (p ˂ 0.05, SNK test)

**
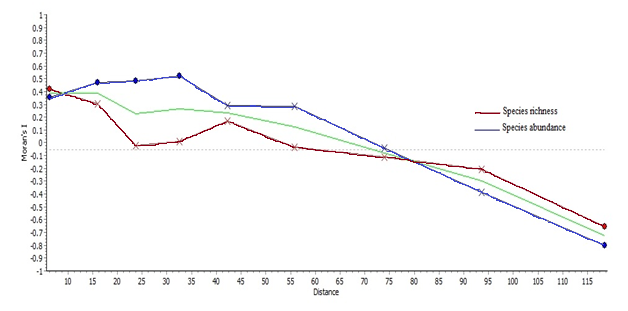
 Fig. S1:** Moran’s I correlogram of termite species richness and abundance in five cocoa agroforestry systems

Fig. S2 Relationship between age of cocoa farms and percent shade cover (r = 0.92)

y = -0.003x^2^ + 0.35x - 2.56
R² = 0.68; p<0.0001; F=18.56

y = -0.003x^2^ -0.22x + 4.78
R² = 0.69; p<0.0001; F=19.60

Fig. S3. Relationship between shade tree canopy cover and richness of termite pest species. Each point represents the mean value of all observation of 10 individual trees per plot.
